# Perioperative management of infected skin defect with poor wound healing in a neonate with Pentalogy of Cantrell associated with single ventricle and ectopia cordis: a case report and literature review

**DOI:** 10.3389/fped.2026.1905614

**Published:** 2026-09-15

**Authors:** Zhiye Yao, Chaoying Zhang, Yuhui Yu, Yumei Liu, Lin Chen

**Affiliations:** Department of Neonatal Intensive Care Unit (NICU), Guangdong Provincial People's Hospital (Guangdong Academy of Medical Sciences), Southern Medical University, Guangzhou, China

**Keywords:** case report, ectopia cordis, incision dehiscence, Pentalogy of Cantrell(POC), single ventricle CHD, skin defect

## Abstract

**Background:**

Complete Pentalogy of Cantrell (POC) with ectopia cordis, full-thickness skin defects, and functionally univentricular physiology is an extremely rare congenital malformation with limited reported management experience.

**Case description:**

We report a male infant with complete POC, including ectopia cordis, a 4 × 4 cm skin defect, and complex congenital heart disease (single ventricle, tricuspid atresia, double-outlet right ventricle, and pulmonary artery hypoplasia) associated with lower sternal defect and omphalocele. Diagnostic workup confirmed the diagnosis according to the Toyama classification; genetic analysis identified no pathogenic variants related to the phenotype. A multidisciplinary staged strategy was adopted. Following targeted antimicrobial therapy for an early local infection, postoperative incision dehiscence resulted in recurrent cardiac exposure. Rather than immediate reoperation, the team elected a conservative approach involving condition-adapted local wound management guided by wound bed preparation principles, alongside optimization of cardiorespiratory function and nutrition.

**Results:**

The exposed cardiac surface achieved complete epithelialization, obviating the need for short-term secondary skin grafting and stabilizing the patient for future staged interventions.

**Conclusion:**

This case illustrates that an individualized, multidisciplinary staged approach—including meticulous local wound care—may facilitate favorable outcomes in selected patients with this life-threatening condition.

## Introduction

The Pentalogy of Cantrell (POC) is a rare congenital anomaly characterized by a spectrum of defects, either partial or complete, involving a distal sternal cleft, an anterior diaphragmatic defect, a midline abdominal wall defect, a pericardial defect, and congenital intracardiac anomalies. The incidence is estimated to be between 1 in 65,000 and 1 in 200,000 live births (1–4). Extreme phenotypes, such as complete POC with a single ventricle, ectopia cordis, and an overlying skin defect, are rarely reported and are associated with a dismal prognosis (5, 6).

The patient in this case presented with all the aforementioned high-risk factors. Through comprehensive multidisciplinary team (MDT) management at our center—particularly the successful management of the skin defect, exposed heart, secondary infection, and subsequent wound dehiscence. The patient survived and was successfully discharged. As there is currently no international consensus on the management protocol for such extreme presentations, we report this case to share management experience of severe POC complicated by skin defect and infections.

## Case report

### Clinical history

A male infant was admitted to our NICU 18 min after birth with multiple congenital anomalies. The infant (G2P2) was delivered via cesarean section at 38 weeks of gestation due to abnormal fetal monitoring. The birth weight was 2,160 g, and Apgar scores were 9 at both 1 and 5 min (1 point deducted for skin color). Upon admission, vital signs were as follows: T 36.6 °C, HR 160 bpm, RR 46 breaths/min, and BP 64/39 mmHg with an oxygen saturation (SpO2) of 88%. Physical examination revealed a head circumference of 32 cm and a body length of 45 cm, accompanied by low-set ears and a webbed neck. A 4 × 4 cm skin defect was observed in the subxiphoid region, with ectopia cordis (Figure 1). Written informed consent was obtained from the patient's legal guardians.

**Figure 1:**
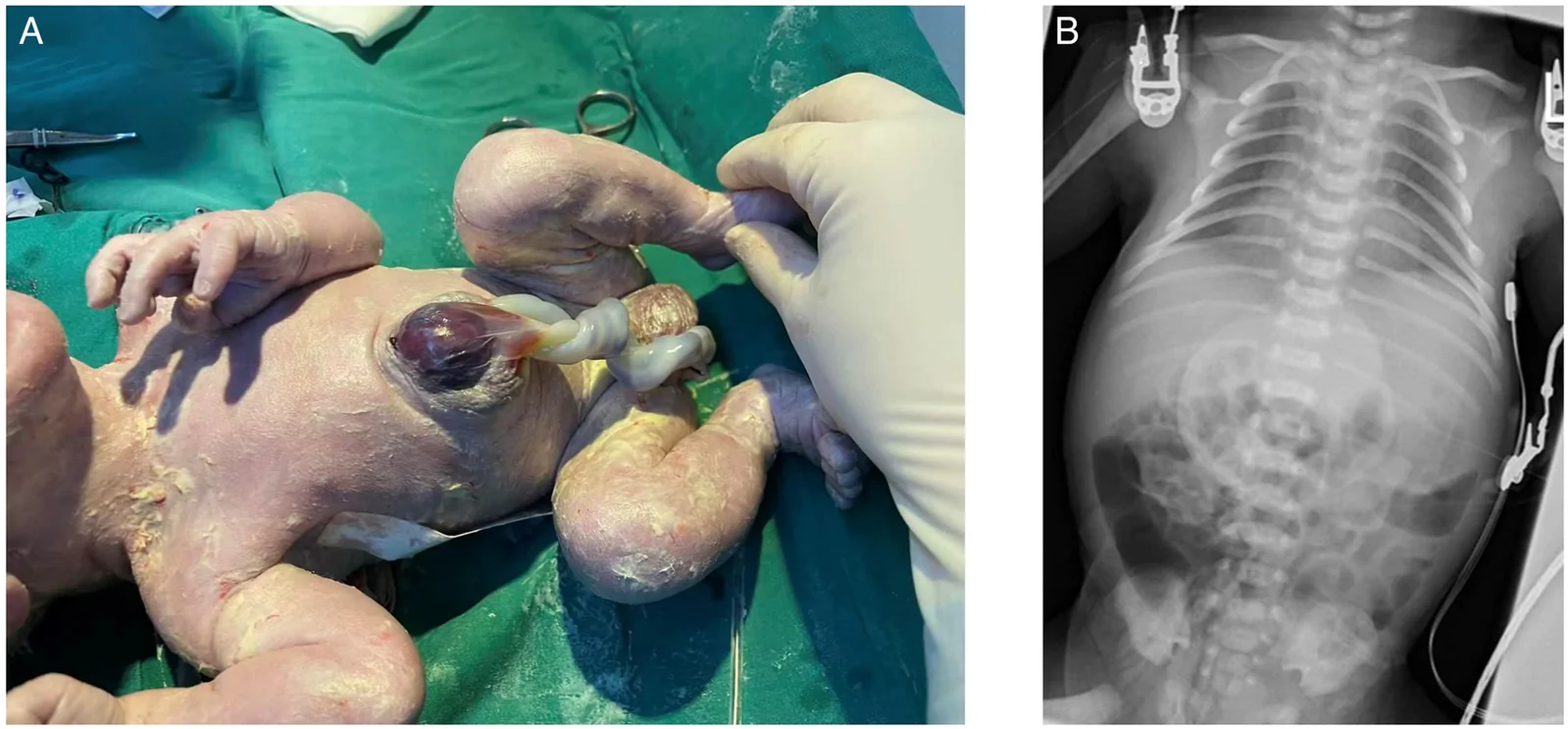
External appearance and the x-ray of the patient. **(A)** Preoperative photograph of the neonate showing ectopia cordis with a large anterior chest wall defect and exposed heart covered by a thin membrane. **(B)** Chest x-ray showing the heart located outside the thoracic cavity (ectopia cordis).

Maternal history showed that the mother was 36 years old and conceived naturally without abnormal pregnancy history. Fetal ultrasonography at 13 weeks’ gestation demonstrated partial extrusion of the fetal heart outside the thoracic cavity and high insertion of the umbilical cord, suggesting POC. Further examinations also indicated complex fetal abnormalities, and termination of pregnancy was recommended to the family. However, the parents refused and were referred to our center, where they requested to keep the baby and receive active treatment after birth. There was no family history of congenital or hereditary diseases. The mother had previously delivered a healthy female infant via cesarean section.

### Diagnostic assessment

Routine laboratory studies, including complete blood count, urinalysis, stool analysis, hepatic and renal function tests, serum biochemistry, electrolytes, and TORCH serology, revealed no significant abnormalities. Cardiac CT with three-dimensional reconstruction demonstrated single ventricle, tricuspid atresia, double-outlet right ventricle, pulmonary artery hypoplasia, collateral formation from the descending aorta, atrial septal defect, and aberrant right subclavian artery. Defects of the lower sternum, ectopia cordis, and omphalocele were also identified, supports the diagnosis of POC according to the Toyama classification (7) (Figure 2). Abdominal and cranial ultrasounds were unremarkable. Chromosomal karyotyping was normal. Whole-exome and mitochondrial genome sequencing revealed no variants clearly associated with the patient's phenotype, although other rare nuclear and mitochondrial variants, including *ALDH5A1* and *UGT1A1*, were detected; neither is implicated in thoracoabdominal wall or cardiac malformations.

**Figure 2:**
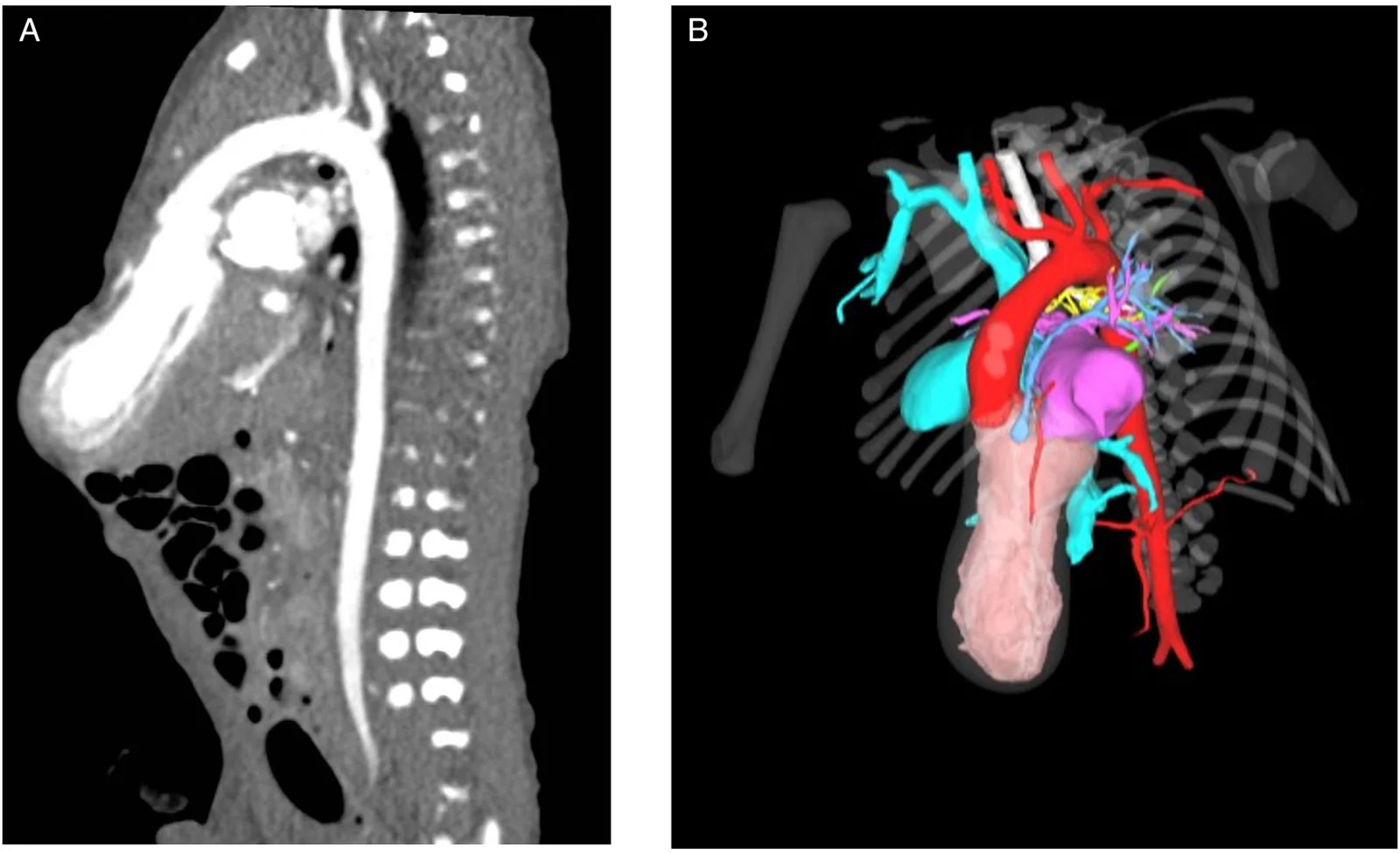
Cardiac CT scan and 3D reconstructed image. **(A)** Sagittal CT image showing ectopia cordis in Cantrell syndrome. **(B)** Volume-rendered 3D CT reconstruction showing the spatial relationship between the exposed heart, single ventricle, and surrounding thoracic structures.

### Treatment

Upon the patient's admission, the MDT established during the prenatal period promptly developed a staged diagnostic and therapeutic plan tailored to the child's specific clinical status. Immediately after admission, the ectopic heart was immediately placed under strict isolation conditions, covered with sterile moist dressings, and protected from mechanical trauma. The infant was nursed in a laminar airflow incubator of the highest available specification within the NICU to maintain thermoregulation and humidity. On postnatal day 4, the infant presented with tachypnea and declining oxygen saturation (from 90% to 80%). Inflammatory markers were elevated, including hs-CRP at 26.3 mg/L, WBC count of 12.84 × 10⁹/L, and neutrophil proportion of 0.76. Given clinical suspicion for external cardiac infection, Piperacillin/Tazobactam (80 mg/kg/dose iv q6 h for 22days) was initiated according to regional epidemiological patterns. Skin cultures subsequently grew hemolytic Staphylococcus, susceptible to the administered antibiotic. The infection was effectively controlled with continued antimicrobial treatment (Figure 3).

**Figure 3:**
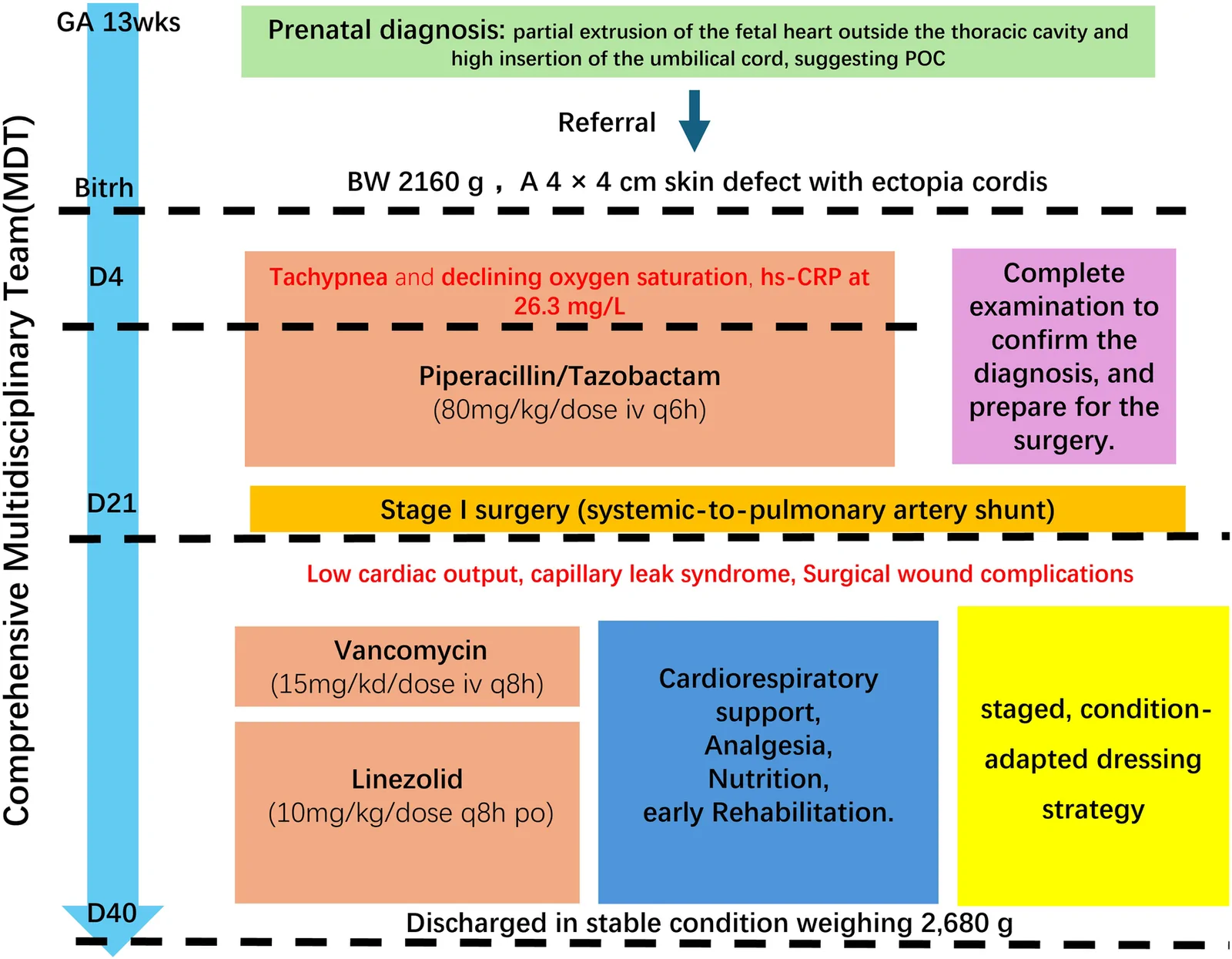
Clinical timeline. Clinical timeline of the patient from prenatal diagnosis through discharge, illustrating staged therapeutic interventions and key clinical milestones.

On day 21, Stage I surgery (systemic-to-pulmonary artery shunt) was performed, accompanied by a free skin graft to cover the defect. Postoperatively, the patient exhibited low cardiac output, capillary leak syndrome, and surgical wound complications including poor healing, violaceous discoloration, necrosis, and dehiscence (Figure 4). Given the critical condition, worsening postoperative infection could not be excluded. While cardiorespiratory support was intensified, antimicrobial therapy was escalated to Vancomycin (15 mg/kd/dose iv q8 h for 7days) following the clinical pharmacist's recommendation. Following infection control, the antimicrobial regimen was de-escalated to oral linezolid (10 mg/kg/dose q8 h po for 21days).

**Figure 4:**
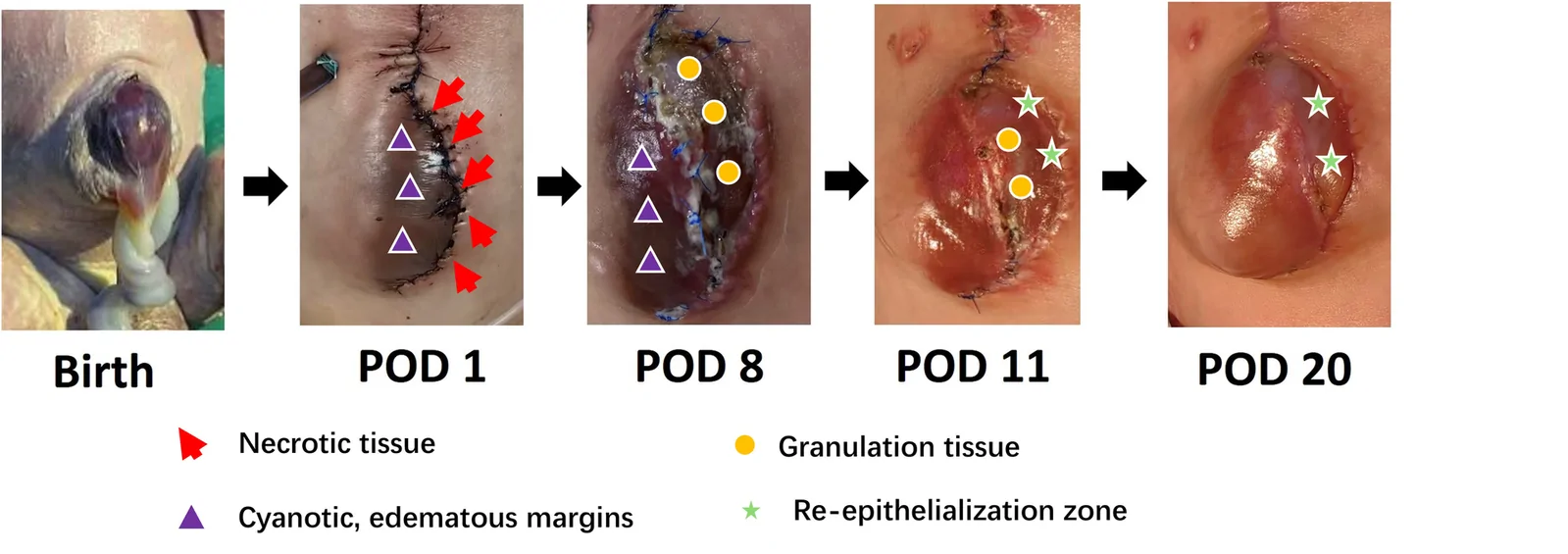
Serial photographs of the surgical incision.

Postoperative wound management was individualized by the MDT, balancing the infant's critical condition with parental preferences, and a localized conservative approach was pursued. Local therapy was staged according to wound bed pathophysiology, and is carried out 1 to 2 times a day. Necrotic tissue was managed with povidone-iodine and mupirocin to reduce bacterial bioburden and control colonization by staphylococci, thereby removing a key barrier to healing progression. At cyanotic, edematous wound margins, mucopolysaccharide polysulfate cream (Hirudoid) was applied to improve microcirculation through its antithrombotic and nitric oxide-mediated vasodilatory effects. Once granulation tissue was established, recombinant bFGF was applied to the wound base to stimulate angiogenesis, fibroblast proliferation, and re-epithelialization, while its anti-fibrotic properties mitigated excessive scarring.

Perioperative management included individualized cardiorespiratory support, adequate analgesia, optimized nutrition, and early rehabilitation. The infant was discharged in stable condition weighing 2,680 g, with ongoing outpatient follow-up for staged treatment.

## Discussion

The survival rate of the complete POC is extremely low, especially when combined with cardiac ectopia and skin defects, which lead to a worse prognosis and significantly increased risk of death (8). In previously reported cases, the cardiac malformations were predominantly atrial septal defects, ventricular septal defects, tetralogy of Fallot, or left ventricular diverticula (9, 10). The main focus has been on prenatal diagnosis and counseling (11), correction of simpler cardiac malformations, and staged repair of thoracoabdominal wall defects (12, 13). However, reports on the management of POC combined with skin defects overlying an exposed heart are rare.

A review of the previously reported cases of Pentalogy of Cantrell with ectopia cordis complicated by overlying skin defects (as summarized in Table 1) reveals that successful survival remains extremely rare, regardless of whether the approach involves surgical skin coverage with cardiac repositioning or conservative management. Among the three conservatively managed cases, only the patient reported by Marcella Schiavone et al. demonstrated a favorable outcome, with stable clinical status and progressive epithelialization of the exposed heart at 5-month follow-up (13). Surgical series have yielded similarly discouraging results, with the notable exception of Mendaluk et al. (14), who achieved successful single-stage repair in a patient with hemodynamically stable, simple cardiac anatomy. This disparity suggests that the complexity and physiological burden of associated cardiac anomalies may be a more critical determinant of early survival than the thoracoabdominal wall defect itself (2). In this case, functional single ventricle with severe pulmonary hypoplasia is traditionally a relative contraindication to aggressive repair. However, our staged approach—focusing on wound stabilization, infection control, and hemodynamic optimization before definitive surgery—shows that survival is feasible even in extreme anomalies, provided the exposed heart is protected and perfusion sustained. Generalizability to similar cases remains uncertain and warrants further study.

**Table 1:** Reported cases of POC with ectopia cordis complicated by skin defects.

Year	Author & Region	Birth Details	PND	CHD	EC Management	CHD Repair	Outcome
2015	Vered Lack from South Africa (5)	Female, BW 2080g, GA 35 wks, NVD	NR	NR	Covered with transparent plastic sheeting and transferred to tertiary center. On DOL 4, artificial pericardial scaffold placed for containment.	NR	Died at 48 h post-op due to Infection.
2015	Teresa Mendaluk from Poland (14)	Female, BW 3,650 g, GA 39 wks, CS	T	VSD, PFO	Single-stage surgery on DOL 2: apex repositioning into thorax, pericardial closure, and abdominal wall reconstruction.	VSD closed at 2 months	Alive and well at 19-month follow-up.
2017	Edward Araujo Júnior form Brazil (20)	Female, BW 2,430 g, GA 34 wks, CS	T	VSD	Surgical correction at 11 h of life: sternal opening, total thymectomy, pericardial resection, bilateral costal arch release, and wide skin flap mobilization.	NR	Died on POD 17 due to septic shock.
2018	Claudiu Mărginean from Romania (19)	Male, BW 3,100 g, GA 39 wks, CS	T	VSD, Severe PS, PDA	Saline-soaked gauze dressing on thoracoabdominal and cranial defects. On DOL 14, defects were repaired.	At 14 days of age, the heart was repositioned into the thorax, a systemic-to-pulmonary shunt, ligation of the PDA	Died 5 h post-op due to hemodynamic deterioration, metabolic acidosis, and hypoxia.
2021	Zlatan Zvizdic from Qatar (4)	Male, BW 2,050g	T	ASD, VSD, moderate PS	Omphalocele covered with saline-soaked gauze and prophylactic ampicillin + amikacin. On DOL 18, primary fascial closure with silo bag placement was performed.	NR	Died 72 h post-op.
2025	Abarham Martadiansyah from Indonesia (15)	Female, BW 1,955g, CS	T	PDA, ASD	Coverage with warm saline-soaked gauze and temporary sterile plastic. Modified Bogota bag placement performed on DOL 2.	NR	Died on POD 5 due to sepsis and heart failure.
		Female, BW 2,800 g, CS	T	TOF, PDA, ASD	Ampicillin and gentamicin prophylaxis. Due to spontaneous pericardial epithelialization, only Bogota bag insertion and omphalocele resection were carried out.	NR	Alive and discharged in stable condition.
2015	Helga Naburi from Tanzania (30)	Term, NVD	F	ASD, VSD	The family cannot afford treatment.	NR	Died suddenly at home at 9 months.
2017	Simon Pius from Nigeria (7)	Term, NVD	F	NR	Exposed heart covered with Sofra-Tulle gauze at birth; warm saline-salt compresses for cord care; Broad-spectrum antibiotics (cefuroxime, gentamicin, and metronidazole).	NR	Died on DOL 11.
2020	Marcella Schiavone from Italy (31)	Female, BW 2,300 g, GA 35 wks, CS	NR	NR	Conservative management with topical eosin application and follow-up.	NR	Alive at 5-month follow-up.

PND, prenatal diagnosis; EC, ectopia cordis; CHD, congenital heart disease; NVD, normal vaginal delivery; CS, cesarean section; BW, birth weight; GA, gestation age; NR, not reported; DOL, day of life; VSD, ventricular septal defect; ASD, atrial septal defect; PDA, patent ductus arteriosus; PS, pulmonary artery stenosis; PFO, Patent foramen ovale; POD, postoperative day; T, ture; F, false.

The postnatal management of exposed ectopic cardiac tissue remains controversial. Unlike omphalocele, where viscera retain the protective covering of amnion and Wharton's jelly, the epicardium in ectopia cordis is completely denuded, rendering it vulnerable to desiccation, mechanical trauma, and bacterial colonization (15, 16). Prior strategies have included conservative exposure with topical care (5, 17, 18), temporary coverage with a Bogota bag (15), and primary surgical closure (14). Conservative management relies on spontaneous epithelialization but subjects the patient to a prolonged window of risk for suppurative pericarditis and sepsis (15). The Bogota bag technique provides temporary barrier protection; however, the experience of Martadiansyah et al. (15) demonstrated that even with prophylactic antibiotics and coverage, cardiac decompensation and sepsis may still occur. Primary surgical closure offers definitive protection but requires favorable anatomy—specifically, a repositionable heart and sufficient soft tissue without excessive tension.

Regarding prophylactic anti-infective therapy, most previously reported cases of POC with ectopia cordis received broad-spectrum antibiotics immediately after birth (4, 19, 20). In the case reported by Abarham Martadiansyah (15), prophylactic antibiotics combined with a protective pouch procedure were implemented for the exposed heart; however, the infant ultimately died of sepsis on postoperative day 5, indicating that prophylaxis does not eliminate infection risk. The decision on anti-infection in this case was individualized and context-dependent, resting on several considerations. First, the exposed epicardium is prone to colonization by skin flora; prophylactic antibiotics may suppress susceptible strains and promote resistance, yet fail to eradicate biofilms (21). Second, early exposure can mask infection signs and delay microbiological diagnosis (22). Third, indiscriminate broad-spectrum use in neonates carries both ecological and individual risks amid rising antimicrobial resistance. The successful infection control achieved in this single case is consistent with the hypothesis that, in centers with intensive monitoring capabilities, a surveillance-triggered targeted strategy may be a viable alternative for carefully selected patients. However, this does not argue against prophylactic use in comparable cases. Therefore, rather than endorsing a universal protocol, we advocate individualized antibiotic decisions in POC, tailored to institutional resources, local epidemiology, and real-time clinical risk stratification.

Postoperative wound dehiscence is a well-recognized complication following surgical repair of POC. Srinivasakumar (23) reported a case of Cantrell syndrome variant in which wound dehiscence occurred after delayed omphalocele repair and was successfully managed with anti-infective therapy and vacuum-assisted closure. However, the majority of such reports involved dehiscence after delayed reconstruction, once the heart and other viscera had already been repositioned into the thoracic or abdominal cavities. Wound dehiscence following primary skin closure over an exposed heart, as observed in the present case, has not been previously documented. Several factors may have contributed to impaired wound healing in this patient, including severe deficiency of thoracoabdominal soft tissue, compromised local perfusion secondary to complex cardiac malformations, persistent mechanical stress from cardiac pulsation, and potential subclinical infection (24, 25).

The treatment for the split incision should be a more aggressive surgical debridement or a conservative dressing change. Considering the critical condition of the child after the surgery and the parents’ desire to minimize the surgical trauma for the child, we adopted a staged, condition-adapted dressing strategy. For areas with marginal necrotic tissue, povidone-iodine combined with mupirocin was used to reduce local bacterial burden and prevent necrotic tissue from serving as a nidus for infection (26–28). Mucopolysaccharide polysulfate was applied to the surrounding edematous and ecchymotic regions to improve microcirculation and limit progression from edema to necrosis (29). Once healthy granulation tissue had formed, growth factors were applied to promote fibroblast proliferation, granulation tissue formation, and epithelial migration (17, 18). Concurrently, aggressive management of postoperative cardiopulmonary insufficiency was undertaken to optimize tissue perfusion, along with enhanced nutritional support, appropriate analgesia, and maintenance of internal homeostasis. Through these multidisciplinary measures, the wound achieved complete epithelialization. This not only provided a stable foundation for the second-stage surgical repair but also obviated the need for successive skin grafting procedures.

## Conclusion

This case underscores the need to integrate local wound care with systemic hemodynamic support in neonates with univentricular physiology and ectopia cordis. Wound healing here is a systemic process dependent on cardiac output, oxygen delivery, and nutrition—not merely a local tissue issue. Previous reports have overlooked the interdependence of these factors. Our staged, multidisciplinary strategy, which prioritizes survival and wound stability over early anatomical repair, offers a potential framework for managing similar extreme POC cases.

## Limitation

As a single-center, single-case report, our findings have limited generalizability. Management in such rare, heterogeneous conditions must be individualized—guided by institutional capacity, team expertise, and patient factors. Future multicenter registries should define evidence-based algorithms and identify which POC subgroups benefit from specific strategies. Another limitation of this case report is the incomplete follow-up data at the time of writing.

## Data Availability

The raw data supporting the conclusions of this article will be made available by the authors, without undue reservation.
